# Beyond Urticaria: Schnitzler Syndrome

**DOI:** 10.4274/balkanmedj.2017.0259

**Published:** 2017-09-29

**Authors:** Ricardo Ruiz Villaverde, Ahinoa Bueno Rodriguez, Daniel Sánchez Cano

**Affiliations:** 1 Clinic of Dermatology, Granada Hospital, Granada, Spain; 2 Clinic of Internal Medicine, Granada Hospital, Granada, Spain

A 48-year-old male was admitted to our outpatient dermatological clinic complaining of a 2 year-evolution of recurrent urticarial rash and arthralgia on knees and elbows. Physical examination showed evanescent hives in the upper third of the chest and back without associated angioedema (Figure 1a). The patient reported intermittent fever (>38.5 °C) in the outbreaks and swollen neck lymph nodes.

Laboratory tests showed a blood count of 12.990 leukocytes with 77.6% neutrophils, erythrocyte sediment rate of 38 mm/h and C reactive protein (CRP) 75.5 mg/L. Bence-Jones proteinuria, autoantibodies (ANA-HEp2, cANCA, pANCA), complement and thyroid profile were within normal limits. Serum electrophoresis revealed a monoclonal IgM kappa component. On the abdominal and thoracic computed tomography (CT) scan, splenomegaly and multiple lymph nodes in the neck and armpits could be observed. Bone marrow aspiration and biopsy revealed 15% of lymphocytes with lymphoplasmocytosis. Histopathological examination of the skin was consistent with neutrophilic urticaria (Figure 2a) and lymph nodes showed follicular hyperplasia and polytypic plasmacytosis (Figure 2b) with polyclonality to IgH/K/L genes.

Treatment with Anakinra 100 mg/day was proposed after a poor clinical response to prednisone at doses of 1 mg/kg/day for 1 month, colchicine 1 gr/day for 45 days and cyclosporin at doses of 4 mg/kg/day for 6 weeks. Evolution after treatment showed substantial improvement with normalisation of the analytical parameters (Figure 1b). No side effects had been reported in the 1 year follow-up. Written informed consent was obtained from the patient to use clinical pictures for academic and research purposes.

Schnitzler’s syndrome, is an acquired auto-inflammatory disease, which was first reported in 1972. It was considered an autonomous entity in 1989 (1). It is characterised by the presence of urticarial rash, recurrent fever, arthritis/arthralgia, and enlarged lymph nodes. Monoclonal IgM may be considered the biological hallmark of the disease.

We should suspect this entity in adult patients, usually older than 40, with a chronic urticarial rash associated with any of the following signs or symptoms: fever, fatigue, general malaise, arthralgia, enlarged liver, or spleen, enlarged lymph nodes, leukocytosis and/or increased markers of inflammation, monoclonal gammopathy and a neutrophilic infiltrate on skin biopsy (2).

Strasbourg criteria should be applied to define definite or probable Schnitzler’s syndrome (3). Our patient complained of two obligate criteria and the four minor criteria defined.

Differential diagnosis should be established with Adult-onset Still’s disease, and Cryopyrin-associated periodic syndrome (especially Muckle-Wells syndrome). Periodic fever syndromes may be considered when we face a patient with the baseline clinical features presented by our patient. Other entities to consider in the differential diagnoses are urticarial vasculitis, cryoglobulinaemic vasculitis, systemic lupus erythematosus and chronic idiopathic urticaria. The prognosis of Schnitzler’s syndrome is linked to the evolution of the lymphoproliferative disorder (15-20%), whether lymphomas, including lymphoplasmacytic lymphoma, Richter-type lymphoma, marginal zone lymphoma, myeloma or Waldenström’s disease. They may appear 10 to 20 years after the onset of the first symptoms.

The treatment of Schnitzler’s syndrome is a real challenge for the clinician. Exacerbating factors should be avoided when possible. In the absence of alterations in quality of life and without the persistent elevation of inflammation markers (CRP <30 mg/L) there are some therapeutic options available: Colchicine 1 to 2 mg/d, non-steroidal anti-inflammatory drugs for flare-ups of joint and/or bone pain, and pefloxacin or hydroxychloroquine 2x200 mg/d. If the clinical course is severe, Anakinra 100 mg/day must be considered.

## Figures and Tables

**FIG. 1.**
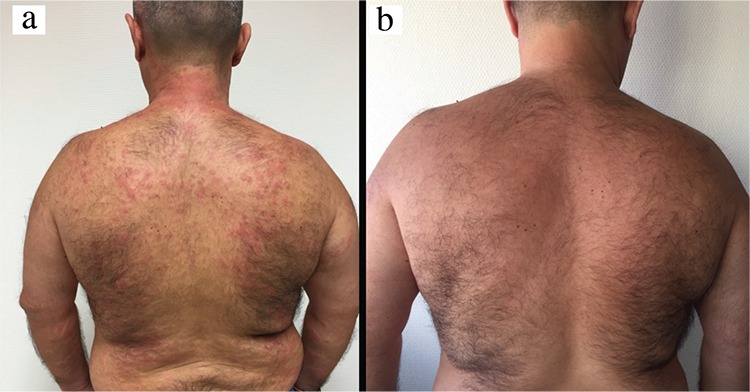
Urticarial rash on the back (a), Clinical improvement after 1 week of treatment (b).

**FIG. 2.**
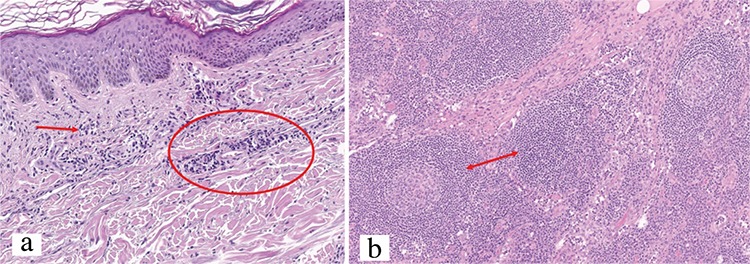
H&E x40: Mixed inflammatory infiltrate (predominantly of neutrophils) in the dermis with no evidence of vasculitis (a), Lymph node biopsy (H&E x40): Follicular hyperplasia and polytypic plasmacytosis (b).
